# interRAI Quality of Life for Mental Health and Addictions: Psychometric Properties and Differences Across Age, Gender, and Service Settings in Brazil

**DOI:** 10.1002/mpr.70040

**Published:** 2025-11-01

**Authors:** Alice Hirdes, Wagner de Lara Machado, Priscila Carvalho Fogaça, Fabiana Rosa de Oliveira Nink, Richard Steiner Salvato, Elton Luiz Ferlin, John P. Hirdes

**Affiliations:** ^1^ Federal University of Rio Grande (FURG) Rio Grande Brazil; ^2^ Pontifical Catholic University of Rio Grande do Sul (PUCRS) Porto Alegre Brazil; ^3^ Lutheran University of Brazil (ULBRA) Canoas Brazil; ^4^ São Lucas University Center Ji‐Paraná Brazil; ^5^ Federal University of Health Sciences of Porto Alegre (UFCSPA) Porto Alegre Brazil; ^6^ Unit of Biostatistics and Data Analysis Hospital de Clínicas de Porto Alegre (HCPA) Porto Alegre Brazil; ^7^ School of Public Health Sciences University of Waterloo Waterloo Ontário Canada

**Keywords:** mental disorders, primary health care, quality of life, rehabilitation centers, validation studies

## Abstract

**Objective:**

Estimate the psychometric properties of the interRAI Quality of Life for Mental Health and Addictions (interRAI QOL) instrument with users of Psychosocial Care Centers and participants of therapeutic groups in Primary Health Care, exploring age, gender, and service settings differences in quality of life.

**Method:**

This quantitative study was conducted with 617 users from Psychosocial Care Centers and Primary Care services in two Brazilian states, Rio Grande do Sul and Rondônia. Data collection was carried out using the interRAI QOL. Confirmatory factor analysis and reliability assessment were performed using McDonald's Omega index. Non‐parametric tests, including Mann‐Whitney and Kruskal‐Wallis, were conducted to compare the Quality‐of‐Life dimensions among participants based on age, gender, and care unit.

**Results:**

The confirmatory factor analysis indicated a good fit for the hypothesized model (CFI = 0.97, RMSEA = 0.08). Reliability was adequate for all subscales according to McDonald's Omega, ranging from 0.71 to 0.88. Gender differences were observed in the well‐being and health dimensions, while all dimensions except support showed significant differences based on age group. The care unit location also revealed significant differences across all dimensions. Participants from Psychosocial Care Center Alcohol and Drugs and from Primary Health Care show better QOL profiles than in other settings and regions.

**Conclusion:**

The interRAI QOL demonstrated adequate psychometric properties and proved to be a valuable new instrument for assessing quality of life among individuals receiving care in the psychosocial care network.

## 1 Introduction

Mental disorders (MD) represent a significant proportion of the global burden of disease, according to evidence from the Global Burden of Disease 2019 (Global Burden Disease 2019; Mental Disorders Collaborators 2022). Moreover, there is no evidence of a reduction in the burden since 1990, despite evidence‐based interventions that can reduce the burden by age, sex, and geographic location. On the contrary, the estimated percentage increase is 48.1% worldwide. Based on estimates from the Institute for Health Metrics and Evaluation (IHME), as part of the Global Burden of Disease Study 2019, the total number of Disability‐Adjusted Life Years (DALYs) per 100,000 inhabitants, with the highest rates in Australasia (30,000), in Latin America (33,000) and in high‐income North America (29,000), serving as a reference for calculating the proportion of disease burden attributable to mental disorders in these regions (Global Burden Disease 2019; Mental Disorders Collaborators 2022). Disability‐Adjusted Life Years (DALYs) represent the sum of years of life lost due to premature mortality and years lived with disability, reflecting the overall burden of disease.

Ferrari and colleagues (Global Burden Disease 2019; Mental Disorders Collaborators 2022) measured the impact and occurrence of 12 mental disorders on a global, regional, and national scale between 1990 and 2019, using various quality‐of‐life‐related metrics such as disability‐adjusted life years (DALYs), years lived with disability (YLDs), and years of life lost (YLLs). The global distribution of DALYs in people with mental disorders (MD) in 2019 by country was similar to the prevalence trends of mental disorders. Depressive and anxiety disorders are considered the leading causes of disease burden (DALYs) and disability (YLDs) worldwide. Brazil is among the countries with the highest DALY rates (Global Burden Disease 2019; Mental Disorders Collaborators 2022).

In addition to MD, alcohol abuse constitutes a public health problem and is associated with an increase in mortality and morbidity rates due to its potential to cause diseases and its relationship with the loss of quality of life (Global Burden Disease 2016; Alcohol Collaborators et al. 2018). In Brazil, alcohol consumption was the sixth risk factor for the loss of disability‐adjusted life years (DALYs) in 2019, accounting for 3,716,649 million (5.69%) DALYs (United Nations 2021). Regarding substance use, whether legal or illegal, the World Drug Report 2021 estimates a 43% increase in consumption in low‐income countries and a 10% increase in middle‐income countries (World Health Organization 2019).

Both common and severe mental disorders are associated with a reduction in quality of life (QoL) (Santos et al. 2019; Vitorino et al. 2022; Berghöfer et al. 2020). Studies on QoL in individuals with mental disorders are among the recommendations of the Comprehensive Mental Health Action Plan 2013–2030 (World Health Organization 2021). The World Health Organization (WHO) Quality of Life Group conceptualizes QoL as an individual's perception of their position in life, within the context of the value system in which they live, and in relation to their goals, expectations, and concerns (WHOQOL Group 1995).

Recent studies on QoL in people with severe mental disorders or alcohol and drug users receiving care in Psychosocial Care Centers (CAPS) and Primary Health Care (PHC) are still scarce in the country. The available studies address the relationship between alcohol use and common mental disorders with patients' QoL in PHC (Santos et al. 2019); common mental disorders (Portugal et al. 2016); QoL of psychoactive substance users, their families, and non‐users in PHC (Moreira et al. 2013); QoL and common mental disorders (Santos et al. 2019; Portugal et al. 2016; Borges et al. 2016); QoL of CAPS users (Assunção et al. 2017); QoL of therapeutic residence inhabitants (Klein et al. 2018); and quality of life in schizophrenic patients (Guedes de Pinho et al. 2018). The CAPS in Brazil are public specialized mental health services that provide community‐based and continuous care for outpatients with severe and persistent mental disorders. The initial access to care for individuals with mental disorders occurs through Primary Health Care, which, in coordination with Psychosocial Care Centers (CAPS) and other specialized services, constitutes the Psychosocial Care Network (RAPS), aiming to ensure comprehensive, territorial, and community‐based mental health care.

Research involving QoL, MD, and addictions generally uses the WHOQOL‐Bref questionnaire (Santos et al. 2019; Vitorino et al. 2022; Berghöfer et al. 2020; Portugal et al. 2016), which is considered the “gold standard” in QoL studies. The results of a study (Portugal et al. 2016) in PHC highlighted that mental health variables, health problems, and financial/structural issues are negatively associated with QoL. However, the authors acknowledge that the selected variables only partially explain the outcome and suggest the need for further investigation using a broader theoretical model, including other variables such as access to health services, their characteristics, and user satisfaction.

The determinants of mental health and mental disorders include not only individual attributes such as the ability to manage thoughts, emotions, behaviors, and interactions with others but also social, cultural, economic, political, and environmental factors, such as national policies, social protection, living standards, working conditions, and community social support (World Health Organization 2021).

Assessing the QoL of individuals receiving care at CAPS and PHC through an instrument specifically designed for this population and based on a broader theoretical model is essential in the context of the Brazilian Psychiatric Reform. An instrument that includes domains related to territory, health services, relationships with the care team, and social support networks aligns with the need to highlight both strengths and areas requiring attention and investment by professionals and policymakers.

In this regard, considering the lack of an instrument tailored for this population, a group of researchers (Morris et al. 2016) from different countries developed an instrument titled interRAI QOL for Mental Health and Addictions (QOL‐MHA), considering the specificities of individuals with mental disorders or addictions. Thus, validating a specific instrument for this population can assist in decision‐making regarding the most effective therapeutic possibilities, as well as in planning interventions, providing appropriate care, and organizing health and mental health services.

### 1.1 General Objective

Estimate the psychometric properties of the interRAI Quality of Life for Mental Health and Addictions (interRAI QOL) instrument with users of Psychosocial Care Centers and participants of therapeutic groups in Primary Health Care, exploring age, gender, and service settings differences in quality of life.

## 2 Method

### 2.1 Study Design

This is a quantitative, cross‐sectional, and methodological study, guided by the STROBE tool. The validation of the instrument was carried out according to international criteria established for instrument validation (COSMIN) (Mokkink et al. 2019). The methodology included five stages: translation, back‐translation, semantic equivalence assessment, expert discussion, and pre‐testing.

After obtaining consent from the authors of the original instrument published in English, two independent bilingual translators translated the instrument into Brazilian Portuguese. Subsequently, back‐translation was performed by two bilingual translators with experience in the healthcare field. Then, two researchers evaluated the versions and suggested modifications that were incorporated into the final version, which was approved by the proposing researcher of the instrument.

Only minor adjustments were necessary in some expressions and in the identification data, considering the particularities of the Brazilian Unified Health System (SUS). The interRAI QoL‐MHA has two versions: a self‐reported version and an interviewer‐administered version. In this study, the interviewer‐administered version was chosen.

The original instrument, published in English, was developed by a research group from the interRAI network (Morris et al. 2016). Validation results with a sample of 2218 participants from six countries (Canada, Belgium, Russia, Finland, Brazil, and Hong Kong) showed that the instrument presents adequate validity evidence for measuring the quality of life of patients in mental health services (Luo et al. 2021; Almeida Mello et al. 2021).

### 2.2 Study Participants

The present study has a sample of 617 adults, 58.1% females, 45.8% with less than 45 years, 46.5% with 45–64 years, 6.7% with 65–74 years, and 1% with 75–84 years. In relation to unit care location, 24.3% were from CAPS III Canoas, 24.3% were from CAPS II regional from Ji‐Paraná, Rondônia, 21.2% were from Primary Health Care (participants of therapeutic groups), 18.9% were from CAPSad Gravataí, and 11.2% were from CAPS II Gravataí, Brazil.

For the most psychometric purposes (e.g., factor analysis, reliability indexes) a rule of 10 participants per item is recommended. Since the instrument consists of 41 items, the minimum recommended sample size for this stage was 410 participants. Adding 10% to account for potential losses and non‐responses, the required sample size was 451 users. Random sampling was used, a technique suitable for homogeneous populations, as in this study.

The inclusion criteria for users of CAPS II, CAPSad, and CAPS III were being in treatment at the service for at least 3 months, not presenting an acute condition at the time of the study, not having moderate or severe cognitive impairment, and not being under the influence of psychoactive substances during data collection.

### 2.3 Data Collection

Data were collected using the interRAI QOL for Mental Health and Addictions (QOL‐MHA) (Morris et al. 2016). The instrument assesses different domains, including personal vision, autonomy and self‐determination, daily activities, family and friends, community, relationships with the care team, privacy, empowerment and support, discrimination and living circumstances, and access to health services. These domains are evaluated through a total of 41 distinct items, in addition to sociodemographic and socioeconomic variables. The questions are structured using a Likert‐type scale, with response options including never, rarely, sometimes, most of the time, and always.

For participant recruitment, prior contact was made with CAPS coordinators and the professional responsible for therapeutic groups in PHC. The interviews were scheduled at the units, at a time previously agreed upon with the local coordination. For data recording, two software versions were developed: an online web‐based version and an offline version.

### 2.4 Data Analysis

Confirmatory factor analyses were conducted using a diagonally weighted least squares estimation method, with adjustment for mean and variance, to investigate the fit of the four‐factor theoretical model. The choice of method is due to the ordinal nature of the items. Factor loadings and intercorrelations between factors were evaluated, and factor loadings *λ* ≥ 0.3 were considered adequate. The fit indices considered to verify the fit of the model were the Tucker‐Lewis fit index (TLI ≥ 0.9) and Root Mean Square Error of Approximation (RMSEA ≤ 0.08, with confidence interval) (Brown and Moore 2012). The reliability levels of the factors were estimated using the McDonald's omega index, which is based on the factor loadings of the items and were considered adequate with values ≥ 0.7. Finally, non‐parametric Mann‐Whitney (Wilcoxon test for independent measures) and Kruskal‐Wallis tests were conducted to compare the levels of the Quality ofLife dimensions between the groups of participants by age, gender, and care unit, adopting a significance level of 5% in all analyses.

### 2.5 Ethical Considerations

This study was performed in line with the principles of the Declaration of Helsinki and in accordance with Brazilian Resolution N^o^. 466/2012 (Brazil. National Health Council 2012). This resolution establishes the guidelines and regulatory norms for research involving human beings in Brazil, ensuring the ethics, dignity, safety, and rights of the participants. The project was submitted to Ethics Committees for Research and approved under the following protocols: CAAE 60213316.9.0000.5349, CAAE 60213316.9.0000.5349, and CAAE 29517319.9.0000.5297.

## 3 Results

Confirmatory factor analysis presents a good fit of a hypothesized model for empirical data, with a Chi‐squared (df) = 870.3 (203), CFI = 0.97 and RMSEA = 0.08 (95% confidence interval = 0.07–0.08). As presented in Table 1, all factor loadings were significantly different from zero and higher than 0.3, ranging from 0.47 to 0.87, showing a moderate to high correlation between items and factors. Factor correlations range between 0.49 (WBH and SUP) and 0.74 (WBH and ACT), showing that factors share at most near 50% of their variance. All omega coefficients were above the 0.7 cutting point, indicating a good reliability for each scale, while the overall omega was 0.91.

**TABLE 1:** Dimension reliability and item's factor loadings for each dimension of QOL.

Dimension (ω)	Items	Standardized factor loading
Well‐being and health (0.87)	A1	0.71
	A2	0.78
	A3	0.78
	B1	0.57
	B3	0.71
	B4	0.70
	C3	0.76
Relationship (0.88)	D5	0.76
	E4	0.73
	E3	0.66
	D3	0.72
	D1	0.73
	D2	0.71
	D4	0.65
Support (0.76)	J1	0.47
	J2	0.55
	J3	0.59
	H1	0.76
	H2	0.87
Activities (0.71)	C1	0.77
	E2	0.71
	E1	0.65

**FIGURE 1:**
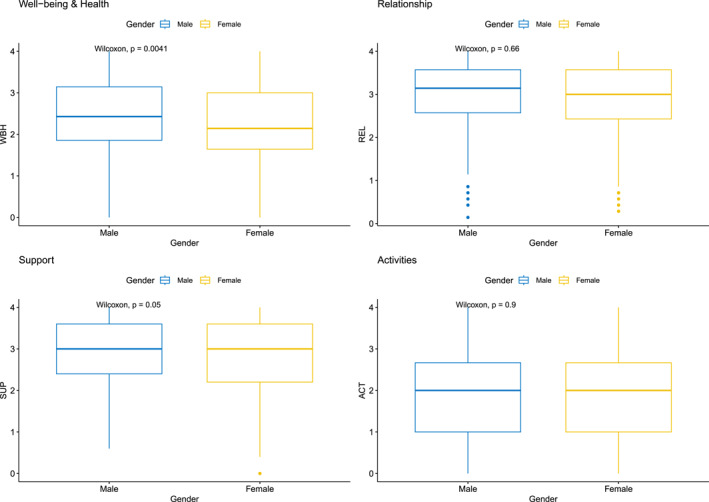
Four dimensions of quality of life for participant's gender.

**FIGURE 2:**
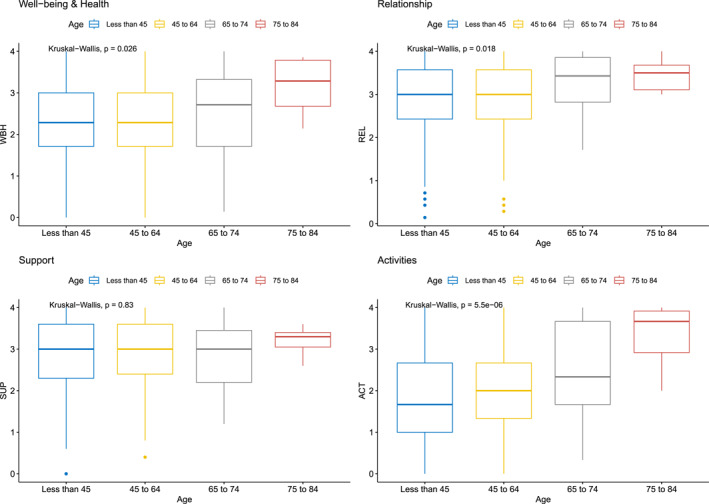
Four dimensions of quality of life for participant's age groups.

**FIGURE 3:**
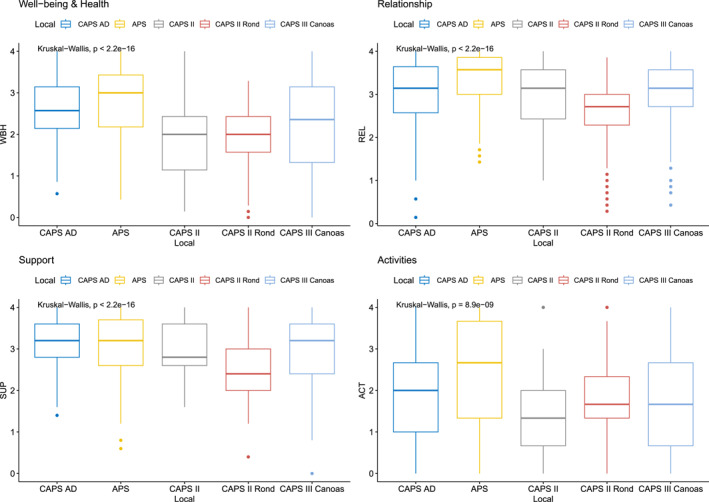
Four dimensions of quality of life for participant's location groups.

Figures 1, 2, 3 show the group comparisons between gender, age groups and care setting, referred as “local”. Most comparisons between groups show significant results for the four QOL dimensions, meaning that those groups variables may can have some influence in QOL levels. No causal inference can be directly made, but it indicates a relevant source of hypothesis generation about how gender, age and socio‐economic determinants can affect QOL indicators, direct or indirectly. For instance, in relation to gender, differences were observable for well‐being and health, and for support levels, with slightly higher levels for male participants. In relation to age, older groups tend to report better, more consistent (less amplitude of scores), QOL than younger ones. Finally, participants from CAPS AD and APS showed better QOL profiles than in other locations/regions.

## 4 Discussion

The interRAI QOL for Mental Health and Addictions (QOL‐MHA) demonstrated excellent psychometric properties and fit to the proposed theoretical model. The theoretical structure was confirmed with excellent fit indices, indicating that the theoretical model has an optimal fit to the data, with no significant unexplained variance remaining, reinforcing the findings of Luo et al. (Luo et al. 2021). Following the psychometric parameters of previous studies (Luo et al. 2021; Almeida Mello et al. 2021), the reliability values for each dimension and the overall score were at a similar effect size level. It is important to highlight that the Support dimension presented borderline values in previous studies, whereas in the current study, it exceeded the cutoff point in the omega index, which is considered more suitable than Cronbach's Alpha for congeneric measures. The factor loadings were adequate, with magnitudes above 0.4, indicating a moderate to high association between the items and their respective factors. Additionally, the correlations between the instrument's dimensions were at most 0.74, indicating discriminant validity among the dimensions, as they had greater specific variance than shared variance.

A study by Rossi et al. (Rossi et al. 2023), conducted to develop mental health indicators in two Psychosocial Care Centers (CAPS), revealed that professionals perceive a limited use of clinical assessment tools and user and family satisfaction evaluations regarding treatment in these services. Consequently, there is a lack of information on the three dimensions of care related to psychosocial rehabilitation: housing, employment, and social networks. These dimensions are directly related to users' quality of life (QoL), and the interRAI QOL‐MHA includes items that address territory, housing, employment, and social networks. This highlights the importance of validating an innovative instrument that encompasses psychosocial rehabilitation constructs from a broader perspective, integrating mental health services, other healthcare services, professionals, and the community environment.

A multicenter study (Luo et al. 2021; Almeida Mello et al. 2021) using the interRAI QoL‐MHA in different countries, including Brazil, found significant associations in various domains, such as the items “financial difficulties” and “work and education opportunities”. These findings emphasize the importance of psychosocial rehabilitation and the need for employment and education opportunities for individuals with mental disorders. Factors that positively influence QoL in individuals with schizophrenia include having a job, not having been hospitalized in the past 5 years, and having social support (Guedes de Pinho et al*.* 2018). Similarly, a study on health‐related quality of life (HRQoL) in outpatients beginning treatment for alcohol or opioid dependence found that being employed, having no comorbidities, and being free of depression were associated with significant improvements in both physical and mental health scores (Simirea et al. 2022).

Another multicenter study conducted in Brazil (Assunção et al. 2017) with mental health service users found that almost half (46.6%) of participants had no individual source of income or relied on labor‐related earnings. This result highlights the barriers to employment opportunities and the negative impact on QoL for individuals with mental disorders. Study (Guedes de Pinho et al. 2018) undertaken with people diagnosed with schizophrenia revealed that 90.3% were unemployed. The results of our study suggest lower quality of life among younger individuals, which may be linked to employment issues, while older participants typically receive retirement benefits or financial assistance from the government. Although direct data on income were not collected, it is reasonable to infer, based on the type of services accessed (e.g., CAPS), that users of the public health system are likely to have limited economic resources. This assumption reflects a contextual understanding of the Brazilian mental health system. A meta‐analysis (Alemu et al. 2024) identified illiteracy, low monthly income, medical comorbidities, and positive and negative symptoms of mental illness as predictors of poor QoL.

The discrimination and living circumstances construct of the interRAI QOL‐MHA showed that users feel accepted and valued by others. However, research indicates that mental health stigma prevents individuals from seeking treatment, leading to reduced QoL and lower social and occupational functioning (Alemu et al. 2024; A. M. Ciobanu et al. 2021; Degnan et al. 2021; Ociskova et al. 2023). Studies (Chen et al. 2023; Almeida et al. 2022) suggest that PHC professionals often lack knowledge about mental disorders, face difficulties in dealing with patients, and exhibit stigmatizing attitudes, creating barriers to recovery. Stigma can also result in microaggressions from friends, relatives, and professionals, manifesting in subtle forms of discrimination, such as social rejection (Barber et al. 2019). In this context, CAPS plays a transformative role, restoring individuals' potential and recognition as political subjects through care strategies aimed at combating stigma (Pires et al. 2023).

Regional differences may be attributed to variations in access to CAPS services. The CAPS Regional in Rondônia serves individuals from 14 municipalities, providing consultations, home visits, and group workshops. However, for individuals living in distant municipalities, individual consultations are the most common form of care, which may explain the results in the staff support recovery and activities domains. Additionally, at both CAPS III and CAPS Regional, the reduction in activities during the first year of the pandemic likely influenced the results. A study (Bonadiman et al. 2017) on the burden of mental disorders in Brazil showed that the North region had the highest proportion of untreated depression cases (90.2%), while the South region had the lowest proportion (67.5%). These regional disparities in access to mental health services and professionals, as well as sociodemographic factors coupled with pandemic‐related challenges, must be considered.

Regarding QoL and gender, research indicates that separated/divorced women (compared to single women) had lower scores in psychological health and social relationships. Additionally, women with lower education levels (high school or below) had lower scores in social relationships and environment domains (Shafie et al. 2021). Coping strategies, substance use disorders, QoL, anxiety, and depression suggest that higher QoL scores were associated with male gender, absence of anxiety or depression, and coping strategies involving lower self‐blame, positive reframing, acceptance, and behavioral disengagement (I. Ciobanu et al. 2020). Women who experienced their first Major Depressive Episode (MDE) during childhood or adolescence show a higher lifetime prevalence of psychiatric disorders and poorer Health‐Related Quality of Life (HRQOL) compared to those with adult‐onset MDE (Jamet et al. 2024). On the other hand, the results of Havnen et al. (Havnen et al. 2024) showed that being male, using pain medication, and having trauma‐related mental disorders were associated with lower health‐related quality of life (HRQoL) scores.

At CAPSad, results were particularly favorable in the staff relationships and staff support recovery domains. The concept of recovery is fundamental to the psychosocial care model in Brazil, as it reflects psychosocial rehabilitation and care quality. Evidence shows that individuals who feel supported in their personal recovery report greater satisfaction with their care (Onocko‐Campos et al. 2017; Slade et al. 2021; Skar‐Fröding et al. 2021; Ricci et al. 2021; Leamy et al. 2016). Social support was identified as a factor associated with QoL among people living with mental illness in Africa (Alemu et al. 2024). Research by Aragão et al. (Aragão et al. 2018) on individuals with Common Mental Disorders (CMD) in PHC found that greater social integration was associated with lower levels of anxiety, depression, and mixed disorders, highlighting the importance of social support networks for these patients. The positive impact of social support on mental health disorders and quality of life has been demonstrated in other studies (Bjørlykhaug et al. 2022; Kakemam et al. 2024).

Although CAPS is highly valued by users and families, promoting autonomy and social inclusion, improvements are needed in employment reintegration programs (Pinho et al. 2018). The WHO QualityRights assessment of CAPSad highlighted its positive impact on users' QoL but identified gaps in income‐generation initiatives and the need to expand 24‐h CAPSad services (Araujo et al. 2023). Income‐generating workshops, based on the principles of the solidarity economy, can strengthen autonomy, facilitate entry into the formal job market, and promote both social and mental health service emancipation (Brasil et al. 2021). In Japan, the Individual Placement and Support (IPS) employment model is widely used in community mental health services and has demonstrated positive vocational outcomes in real‐world settings (Yamaguchi et al. 2022; Igarashi et al. 2023).

However, the validation of an instrument, Avalia‐CAPS, with professionals revealed that three dimensions—outcomes, autonomy (the ability to perform daily activities), and team integration (learning and crisis management)—showed unsatisfactory results. The lowest mean scores were found in coordination with PHC and other services within the health network, as well as intersectoral collaboration (Rocha and Zanardo 2022). These findings highlight the need to train teams to promote user autonomy and improve the coordination of the Psychosocial Care Network (RAPS).

The spatial dimension of psychiatric reform, a new construct in the field of mental health and public health, advocates for the creation of spaces that foster relationships and subjectivities within urban environments (Paladino and Amarante 2022). This concept resonates with the pioneering work developed since the 1990s in Primary Health Care (PHC) in one site. The participants in this study were involved in therapeutic groups and developed a strong bond with the psychologists facilitating these groups, which may explain the positive results across all domains of the QoL‐MHA.

Beyond the therapeutic groups conducted in PHC units, psychologists also utilize non‐conventional physical spaces, such as community centers and churches, as well as non‐physical spaces, such as parks and plazas, to provide care and promote health. This approach redefines the “therapeutic setting”, expanding it to include the city and its surrounding region, with their multiple possibilities. Additionally, users have the option to participate in groups outside their assigned PHC area, respecting their desire for anonymity and privacy. This factor may explain the superior results in all analyzed domains, compared to CAPS.

Venturini (Venturini 2016) argues that deinstitutionalization is more than a transformation in care and therapeutic approaches—it is a process that occurs within the community. Thus, rehabilitation—or, as the author proposes, “habilitation”‐ takes place in the city, through relationships, subjectivity, equality, individuality, solidarity, and diversity. In this sense, the relationships that the community and professionals establish with the patients can serve as spaces to produce life and quality of life, considering that various dimensions of the interRAI MHA instrument are mediated by relationships established with service professionals, family, friends, and the community.

Thus, considering that the Social Determinants of Health (SDoH) also include environmental factors, such as national policies, social protection, living standards, working conditions, and community social support, it can be inferred, based on the dimensions analyzed, that users feel supported by professionals, family members and by community. However, opportunities for work and education are limited, as has already been demonstrated in other research (Almeida Mello et al. 2021; Pinho et al. 2018; Araujo et al. 2023; Brasil et al. 2021). Regarding age, older adults presented better quality of life outcomes than younger individuals. Older adults are generally retired, which ensures a minimum income, whereas young people only receive benefits from the government if they have a more severe disorder. This means that even if these individuals do not have a formal job or source of income, they receive resources for their maintenance, even if minimal, from official government agencies. In Brazil, three key dimensions—housing, employment, and social networks are fundamental to psychosocial rehabilitation. As these dimensions are also SDoH, it is essential to strengthen this triad, especially among users of Psychosocial Care Centers (CAPS).

Some hypotheses can be raised, such as the fact that all participants are cared for by services of the Brazilian Unified Health System (SUS), which guarantees free care for a population with lower income. Coupled with lower income, people with mental disorders face significant barriers to education and reduced opportunities for labor market participation, including competitive employment and sustained work integration (Adamus et al. 2025; Franke et al. 2024; Evans‐Lacko et al. 2022). Having a mental disorder also affects the possibility of studying, considering that many mental disorders emerge in late adolescence, generating prejudice and self‐stigma. A family member with a severe mental disorder often requires a family caregiver. Thus, a vicious cycle is established: lower family income, reduced educational opportunities and, consequently, limited access to formal employment.

## 5 Conclusion

CAPS plays an essential role in Brazil's Psychiatric Reform, alongside the decentralization and integration of mental health care in Primary Health Care (PHC). In this context, the validation of the interRAI QoL‐MHA, based on a broader conceptual model, provides a specific and psychometrically robust QoL instrument, which is highly relevant to the Psychiatric Reform framework. The instrument includes items that focus on SDoH, such as community, social support, access to health services. In this sense, it is important to highlight the relevance of the instrument for contexts involving vulnerable populations.

### 5.1 Relevance for Clinical Practice

The validation of a quality of life instrument specific to mental disorders and addictions is particularly valuable for countries that have undergone or are currently undergoing psychiatric reform and the transition to a community‐based model of mental health care. The interRAI QoL‐MHA, will also provide key indicators on care quality, individual experiences with services, and service or program evaluations. In this sense, the instrument is an important tool to support clinical interventions, and the development of individualized therapeutic plans tailored to each person.

### 5.2 Strengths and Limitations

A strength of this study is that we had access to a large dataset (*N* = 617) from four Brazilian mental health care organizations and from PHC. The population of the four CAPS are representative of the population treated in this mental health setting in Brazil. To address potential bias, the participants were from different types of CAPS and states.

One limitation of this study was the lack of sociodemographic data, such as income and education level. It is suggested that future research incorporate these variables and investigate the quality of life of CAPS users on a larger scale, as well as that of participants in income‐generating workshops, considering the existence of such projects in different regions of Brazil. A potential bias relates to the fact that all study participants were receiving care through public health services, which generally means they have lower income levels. However, it is important to emphasize that, according to the principles of Brazil's Unified Health System (SUS), access to services is universal for any Brazilian citizen.

## Author Contributions

**Alice Hirdes:** conceptualization, methodology, supervision, Project administration, writing – original draft, writing – review and editing. **Wagner de Lara Machado:** validation, formal analysis, writing – review and editing. **Priscila Carvalho Fogaça:** data curation, investigation, supervision, writing – review and editing. **Fabiana Rosa de Oliveira Nink:** data curation, investigation, supervision, writing – review and editing. **Richard Steiner Salvato:** data curation, investigation, visualization, writing – review and editing. **Elton Luiz Ferlin:** software, data curation, visualization, writing – review and editing. **John P. Hirdes:** visualization, validation, writing – review and editing.

## Consent

Informed consent was obtained from all individual participants included in the study.

## Conflicts of Interest

The authors declare no conflicts of interest.

## Data Availability

The data that support the findings of this study are available on request from the corresponding author. The data are not publicly available due to privacy or ethical restrictions.
